# Universal Principles of Depicting Oneself across the Centuries: From Renaissance Self-Portraits to Selfie-Photographs

**DOI:** 10.3389/fpsyg.2017.00245

**Published:** 2017-02-21

**Authors:** Claus-Christian Carbon

**Affiliations:** ^1^Department of General Psychology and Methodology, University of BambergBamberg, Germany; ^2^Bamberg Graduate School of Affective and Cognitive SciencesBamberg, Germany; ^3^Research Group Ergonomics, Psychological ÆstheticsGestalt, Bamberg, Germany

**Keywords:** selfie, art history, self-portrait, Albrecht Dürer, Renaissance, painting, photograph, human condition

## Abstract

Selfie-photography is generally thought of as a cultural mass phenomenon of the early 21st century, inseparably related to the development and triumph of the smartphone with integrated camera. Western culture, however, has been highly familiar with self-depictions since the Renaissance days. Putting the contemporary selfie into this historic context covering more than five centuries of cultural development from Dürer's (1500) famous “Self-Portrait at 28” (also known as “Selbstbildnis im Pelzrock”) to today's Instagram galleries allows for identifying central parallels concerning the technical and social antecedents as well as common underlying psychological factors and shared properties of different kinds of self-depiction. The article provides an overview of the types of contemporary photographic selfies and compares them with painted self-portraits. Finally, this historic perspective leads us to the insight that self-portraits as well as selfies are both referring to nothing less than the “conditio humana.”

When Albrecht Dürer signed his famous self-portrait with his imposing monogram “AD” in 1500 (see Figure 1) he did not just finish a masterwork, but set the foundation for a quite persistent cultural phenomenon: the phenomenon of self-depiction or, as we would call it today, the selfie. In the following article I will show that Dürer and other great self-portraitists expressed themselves using universal principles that are also reflected in today's selfie-photography. Taking a historic perspective I will compare self-portraits and selfies in order to elaborate on differences and commonalities, finally showing that these different kinds of self-depiction are referring to nothing less than the “conditio humana”—specifically, the basic cognitive and affective human needs.

**Figure 1:**
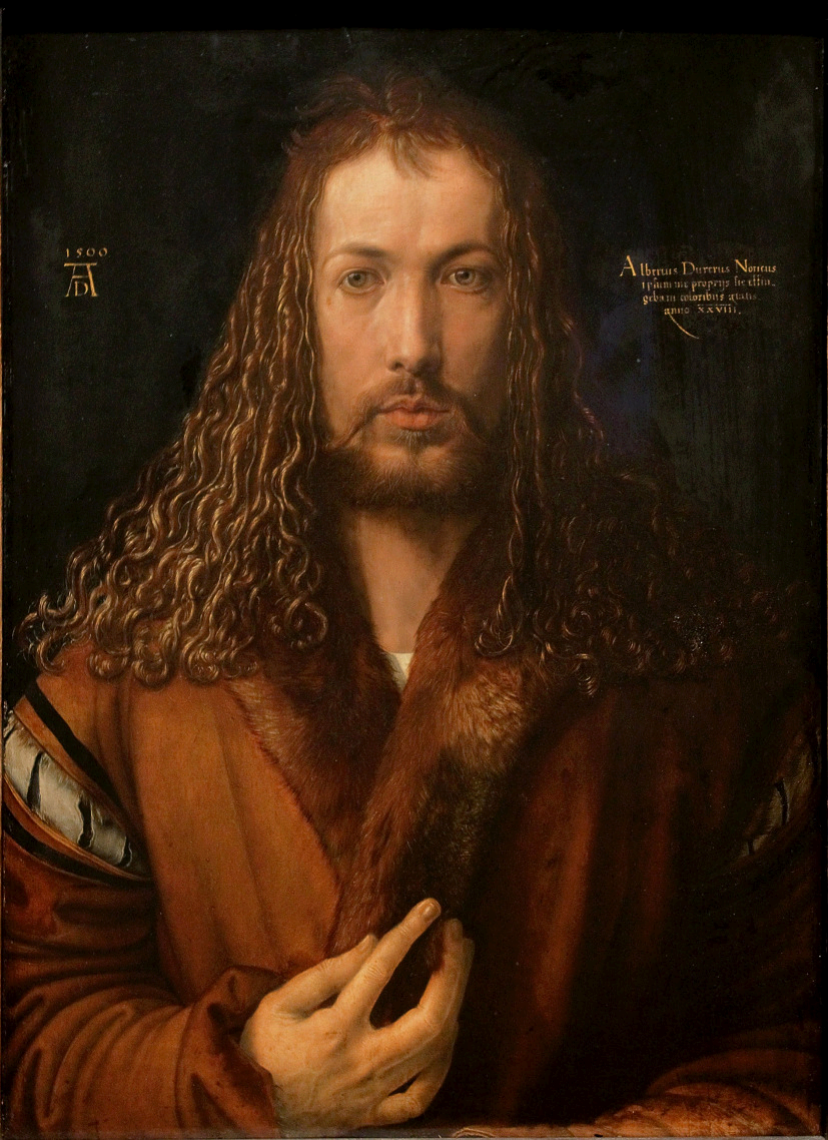
**Albrecht Dürer's “Self-Portrait at 28” from the year 1500, also known as “Selbstbildnis mit Pelzrock”—this picture and its reproduction are in the public domain (Creative Commons CC-BY license)**.

## I am unique, am i not?

Comparing contemporary selfies and historic self-portraits such as Dürer's Renaissance masterpiece from 1500, we first of all notice a number of clear differences, among them concerning the production process, the medium itself and typical compositions: Self-portrait paintings are created following a sophisticated plan or concept demanding a serial production process ranging from composition and preliminary sketches to colorization and final varnish. Apparently, the effort invested in a self-portrait is quite high, and the same is true for the monetary costs, as the used materials (color pigments, large canvases, or wooden panels) are typically quite expensive. Selfies, in contrast, are produced (i.e., taken) within seconds, usually by means of the deficient add-on camera of a smartphone equipped with a strongly distorting lens and under suboptimal lighting conditions. Compositional factors do not seem to be taken into account, and a special preparation is not required. Most often, selfies are the product of a spontaneous intuition, feeling, or idea, which distinguishes them not only from painted self-portraits but also from professional portrait photography that usually follows a complex set of established compositional principles (Bruno et al., 2014), such as the principle of eye centering (Tyler, 1998), for example.

The differences in production process and costs imply another distinction of self-portrait and selfie which is related to limitation and limitlessness, respectively: While the number of self-portraits an artist can create during his lifetime is rather small (e.g., although considered as being notorious for depicting himself, Diego Velázquez only created about four self-portraits out of his entire oeuvre of approximate 120 paintings; Dürer, who is also known for his self-referential artworks, also painted “only” three self-portraits in oil^1^. There are other cases who are truly prolific self-portraitists; for instance, Vincent van Gogh who produced more than 43 self-portraits. Similar inclinations toward painting themselves were found in the oeuvres of Egon Schiele, Edvard Munch, and Frida Kahlo^2^, see Belle (2000). The number of selfies one can take, in contrast, is hardly restricted, except for limits set by factors such as storage capacity of the technical device that is used. Painting a self-portrait (or the repro of a self-portrait) takes weeks, but taking a selfie takes merely the blink of an eye and within another blink, each selfie can easily be copied and distributed to the other side of the world via digital social networks, for instance. Additionally, we should not forget that even the single shot of a photographic selfie is rarely one single shot only: selfie-ists often undertake a great number of attempts to reach their goal of presenting themselves in the desired way). Taking this into account, it is quite sensible to ascribe the attribute of being original and unique to an artist's self-portrait, while such an ascription is rather out of question with regards to selfie photographs.

## The essence of self-depictions

So far, self-portraits and selfies do not seem to have too much in common. But leaving the level of superficial comparison and entering a more phenomenological reflection, we will see the differences vanish. The important question to ask here is: What is the purpose of self-depiction? What exactly is the individual's idea behind painting or photographing oneself?

Essentially, self-portrait and selfie are both based on the idea or wish to freeze, to maintain or to document a fluctuating but significant slice of life. So the primary purpose of these types of self-references is (about) the same even if the quality of execution may be different; namely planned and enduring in the case of the self-portrait but spontaneous and intuitive in the case of the selfie. The notion of “quality” does not evidently mean that selfies are “inferior” to paintings; quality is meant here first of all as being of a “different quality.” Here, it is necessary to ask the next question already: Is the typical selfie-photographer's intuition-based spontaneity really so different from the artist's well-planned behavior?

Our intuitive behavior is not actually based on some amorphous, arbitrary and unintelligent procedures but it condenses our knowledge on a topic, on achieving a specific goal. If people use their intuition when taking a selfie, they use their “intelligence of the unconscious” (Gigerenzer, 2007). This means they express by intuition something that they could hardly or not at all explain in an explicit way. Usually, taking a selfie means to follow the wish to express something special—“selfie-ists” want to create or invent themselves, they want to refer to themselves and they want to boil their inner status, mood, feelings and cognitions down to an essence^3^ (Freeland, 2010). The art historian Ernst Gombrich wrote in his influential book “Art and Illusion” (which is a key text for art historians as well as perceptual scientists) about the difficulty in coming up with such an essential picture, especially if we use photography:

“In fact only a few snapshots will so satisfy us. We dismiss the majority as odd, uncharacteristic, strange, not because the camera distorts, but because it caught a constellation of features from the melody of expression which, when arrested and frozen, fails to strike us in the same way the sitter does. For expression in life and physiognomic impression rest on movement no less than on static symptoms, and art has to compensate for the loss of the time dimension by concentrating all required information into one arrested image”

(Gombrich, 2002, p. 292).

Within this view, self-portraits or selfies are not just a reference to “pathological narcissism” (Hall, 2014, p. 276), instead they transport the essence of the inner states of a person. As we do not have adequate access to the inner states of the sitter—even if we are portraying ourselves—intuition seems to be a promising avenue for giving these hidden states a readable expression. Self-portraits in the established art-historical sense also want to make explicit the inner states (Billeter, 1986; Freeland, 2010)^4^—at least the way we interpret them (Wegner, 2003)—to the outer world, and they also have to rely—at least in the initial phase of creation—on intuition: Ernst Gombrich described this quite elegantly when he stated that in the process of generating a portrait “making will come before matching, creation before reference” (Gombrich, 2002, p. 85). The used methods to execute the work in the following might be much more sophisticated, but the general program remains quite similar. In the end, just one single picture is shown portraying the (inner) complexity of an individual (Gombrich, 2005)^5^. This does not exclude that self-portraits are also often used to advertise the artist's skills, to practice the difficult technique of painting herself/himself or just to make clear that the artist is relevant enough to be portrayed. At least with a second, more analytic view on all these rationales it is quite clear that self-portraits also reveal something about the artist who initiated and created the pictures. The web initiative *The Self-Portrait Experience* (selfportrait.eu) sums this up concisely:

“A self-portrait is our inner image, our private image. It is generally produced in a longer lapse of time, in a situation centered on the creative process. It springs from the inner life of the author, who is also subject and spectator. He does not control the image, on the contrary, it's the creative process which allows the unconscious to speak with the language of art. The self-portrait is a profound dialog with oneself, guided by the author's vulnerability.”

As already mentioned above, intuition might be a promising source of returning such an inner dialog to an explicit expression. In the view of Gigerenzer (2007), intuition is not a deficient, odd-working, and superficial system but a very well adapted mechanism in order to cope with the complexity and uncertainty of the world around. In this sense, intuition is not just a fast but also a powerful and adequate mechanism. This view is quite contrary to the perspective of Kahneman (2003). In his Prize lecture held when awarded with the Nobel Memorial Prize in Economic Sciences in 2002, he proposed that intuition belongs to the two-system model's *System 1* which is characterized by “systematic errors” (p. 450). Still, Kahneman makes clear that intuitions are capable of dealing with complex problems fast and in parallel, automatically, and associatively. System 1, according to Kahneman, can learn new associations only very slowly, but can apply associated routines fast. That is why intuitions will lead to reliable and systematic outcomes depending on the already learnt associations. In sum, both views, although being contrary to each other in many respects, assume that intuition leads to systematic interpretations and behavior based on them.

## The birth of historic and contemporary self-depiction

**Figure 2:**
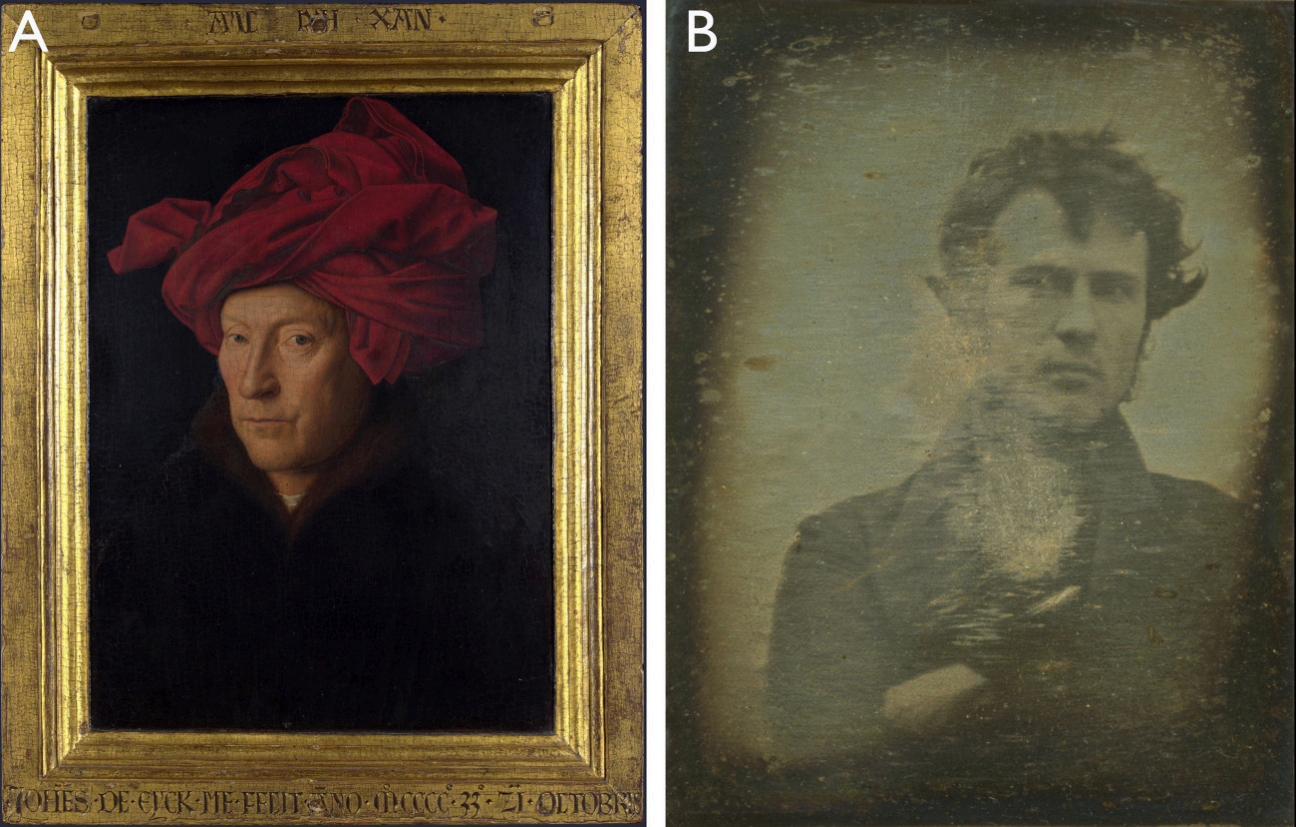
**(A)** “Portrait of a Man” by Jan van Eyck from the year 1433. The works of art depicted in this image, and the reproduction thereof, are in the public domain worldwide. The reproduction is part of a collection of reproductions compiled by The Yorck Project. The compilation copyright is held by Zenodot Verlagsgesellschaft mbH and licensed under the GNU Free Documentation License. **(B)** An early photograph (daguerreotype) made in November 1839 (Sachse, 1893) by Robert Cornelius depicting himself is widely referred to as the first selfie in world history according to many sources (e.g., Newhall, 1949; Hannavy, 2005)—it seems that it is at least the first self-portrait made by daguerreotype-processing. The picture is in the public domain.

Early self-portraits emerge in the early to middle Renaissance era, around the beginning of the 15th century (Gombrich, 2005). Some sources have identified the “Portrait of a Man”^6^ painted by Jan van Eyck in 1433 as the world's first self-portrait (see Figure 2). Whether this specific painting or even an earlier one was literally the first one is not essential here, but at some point of art history, around 1400, painters started to depict themselves—note: we can, of course, not exclude the fact that there were other developments in art which have not been documented, but based on the still-existing artworks, the 15th century seems to be a rough estimation of the point at which self-portraits became a general *sujet* of art history. This was not only done for the sake of having an image of their own, but to express a certain state of their own to *others*, to the *public*.

The emergence of self-portraits is closely related to the (re-)introduction of linear perspective in the arts and to technical inventions and advances such as the engineering of the first high-quality mirrors, e.g., mirrors with coating glass and a tin-mercury amalgam in Germany during the early phase of Renaissance (Melchoir-Bonnet, 2001). The breakthrough for a broader market was the establishment of a center for the production of tin amalgam mirrors in Venice around 1507 (Hadsund, 1993). Such highly sophisticated reflecting devices allowed artists to get a “clear” image of themselves before and when painting their own likeness, especially because they were much brighter and larger^7^ and also less distorted than those which had been available before (Hadsund, 1993). Even before these sophisticated mirrors became available, art history refers to some single exemplars of self-portraits, but the generic genre of “self-portraits” had not yet been developed at this point (Harbison, 1995)—we are referring to self-portraits having formed an own and venerated generic artistic category since the 16th century (Hall, 2014). Later on, the development of classic photo cameras in the 1830s (Hirsch, 2000) made it possible to make self-photographs, although this was far more complicated than today, firstly, as exposure time was extremely long (often more than 10 min), and secondly, as the photographer could not see his own depiction while taking the photo—still some of the very early attempts are just stunning, such as the very first selfie in world history^8^: When Robert Cornelius made a photograph of himself, he created a lively, dynamic, off-centered and very contemporary looking selfie (see Figure 2)—one reason for this modern touch might be the usage of exterior light which could have effectively reduced the exposure time drastically (Hannavy, 2005)^9^, but mainly this appeal seems to be emerging from the non-symmetrical view and the specific combination of gaze to the right and head direction to the left. Importantly, the limiting factor exposure time was markedly reduced over the following 100 years by using more light-sensitive photographic emulsions. It should be noted in this context that the inventor of the stereoscope, British physicist Charles Wheatstone (1802–1875), was probably not only a keen photographer but was also the creator of the first self-portrait of a scientist ever (Wade, 2014)— for a depiction of this self-portrait as well as for depictions of other selfie-and-portrait milestones, e.g., a very early stereoscopic self-portraits, see Wade (2016, p. 272). The essential step toward perceiving oneself while taking a photographic self-portrait required further radical inventions later on, such as CMOS active pixel sensor technology in the 2nd half of the 20th century (Prakel, 2009). In the 1990s, digital cameras were developed that were equipped with first displays that allowed to instantly view the photographed pictures. With the arrival of front-view cameras in cell phones and smartphones in the early 2000s true selfie-photographing was possible for the first time (Wheen, 2011): People were now able to directly control the picture and to optimize the statement they wanted to convey, e.g., to show-off, to trigger empathy or just to document themselves in specific social contexts.

## The case of Albrecht Dürer's self-portraits

**Figure 3:**
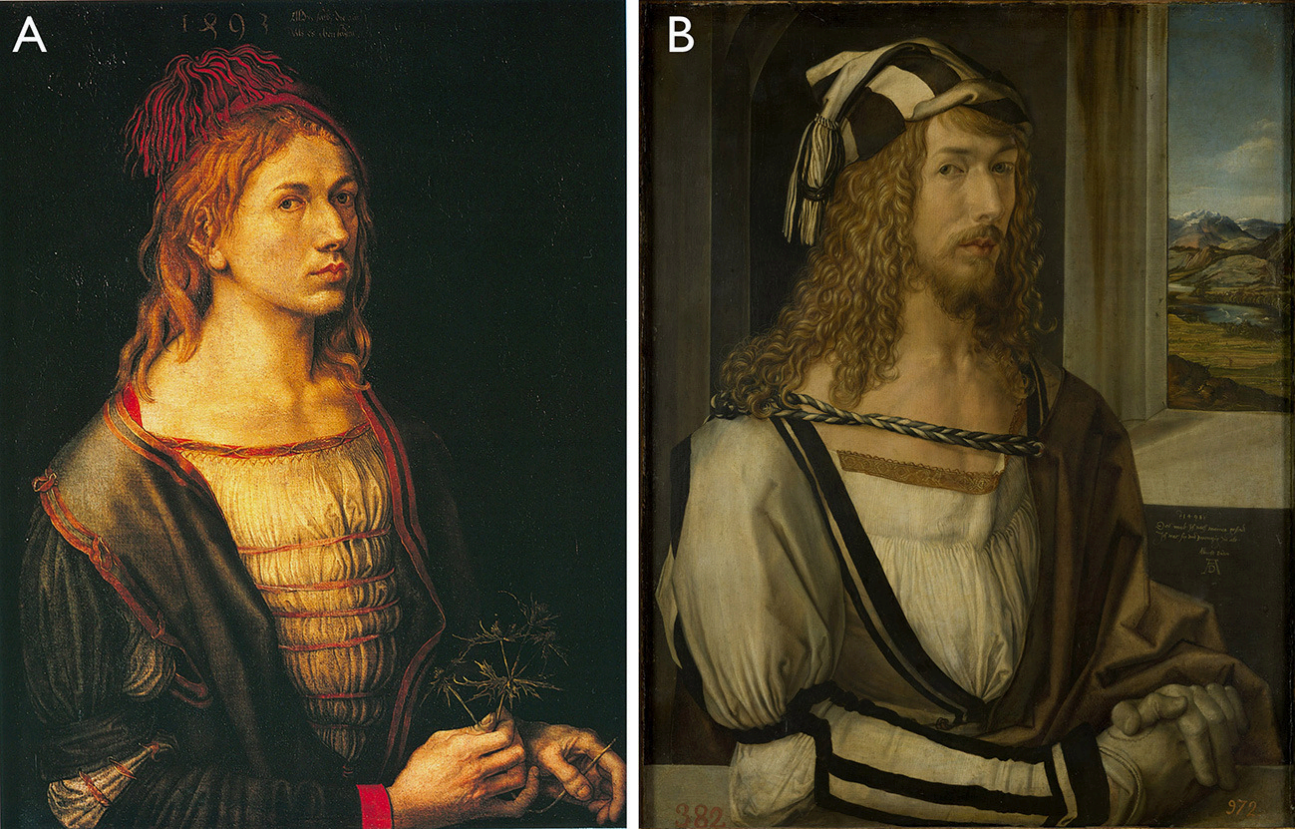
**(A)** Dürer's self-portrait from 1493, oil on parchment mounted on canvas. **(B)** Dürer's Self-portrait from 1498, oil on panel. In some publications, the two paintings are also denominated as the *Louver Self-Portrait* (1493) and the *Prado Self Portrait* (1498); so like with Leonardo's most famous portrait of the Mona Lisa, these two museums share two painted versions of the very same person (cf. Carbon and Hesslinger, 2015). Both pictures belong to the public domain work of art.

Albrecht Dürer's self-portrait from 1500, fully entitled “Self-portrait at 28 years old wearing a coat with fur collar” (German: “Selbstbildnis im Pelzrock”) is a fine example for a self-depiction with a strong statement. This self-portrait was not Dürer's first self-depiction—at the age of 13 years, he already produced a self-referential depiction, drawn with a silverpoint; this early portrait was followed by a series of further self-depictions with varying techniques such as pen and dark brown ink, oil on parchment and oil on panel (see Figure 3). All of these earlier works are of a certain quality resembling nowadays selfie-photographs as they look like some spontaneously taken moments in time. The presence of the depicted person is very strong.

Compared to the spontaneous and lively character of the earlier self-portraits caused by the combination of a ¼ view and direct eye contact with the beholder, Dürer's self-portrait from 1500—composed as a frontal portrait—is clearly less selfie-esque. However, in terms of its symbolic or communicative core this later masterwork anticipates specifics of the contemporary selfie. First of all, Dürer wants to make clear that he is not just another painter, but belongs to a certain and very extraordinary class of people (Koerner, 1990)—contemporary selfies are used to express similar statements: the selfie-ists wants to present themselves as unique and distinct persons, else it would not make sense for them to depict themselves without being pressured to do so. Dürer underlines his message to the recipient using several paraphernalia of high status, e.g., his collar is made of fur from the weasel which was exclusively worn by the elite in the Holy Roman Empire of those days, and even implicated that the wearer was electable for the city council (Bulst et al., 2002). Interestingly enough, Dürer was neither rich nor did he officially belong to the elite of Nuremberg when he worked on this painting (Zitzlsperger, 2012). His economic success actually started only after his 2nd visit to Venice in 1506 (Eaton, 1882).

Until the Renaissance era, painters—artists in general—did not have a specific prestige, because the separation of craftsman and artist had not been solidly established yet. The fundament for this emancipation process was laid by Alberti's influential theoretical book on architecture (*Re Aedificatoria*, i.e., “On the art of building,” completed in 1452), which introduced the concepts of minor vs. major arts (Alberti, 1988). In Germany, this emancipation process took a bit longer and Dürer is one of the prominent protagonists who finally broke with the convention that people who made artwork were just another kind of craftsmen. In fact, he propelled the idea that his artworks were created by extraordinary hands, led by an ingenious mind and inspired by heavenly sent ideas (Hall, 2014). With Dürer, the ingenious “Renaissance man,” the true and pure artist with the aura of a superstar, entered the Northern hemisphere. His self-confident aspiration found expression in his highly symbolic self-portrait signed with “1500 AD” that has preserved its (super)lively quality over the centuries and still puzzles and impresses us today.

Religious references can already be found in Dürer's 1493 self-portrait, where he holds an *eryngium* (a thistle) which is a clear reference to the passion of Christ (Zirpolo, 2008). The 1500 self-portrait, however, goes an essential step further: here, Dürer does not content himself with a mere reference to Christ any more, but downright metamorphoses himself into a depiction of Christ. His stylish golden curls, symmetrically arranged, his enigmatic gaze indicating presence (direct gaze at the viewer) and transcendence (looking through the viewer toward infinity) at the same time, and his dignified hand gesture referring to gestures known from Early Christian iconography (Koerner, 1990) underline his, Dürer's, extraordinary status of being a genius, a true creator presented to the world in this self-portrait. Here, we find Dürer in the tradition of the *divino artista*, the artist who creates just like God, the ultimate creator. His work is not about painting, but creating. Importantly, Dürer's reference to God is not at all to be interpreted in a blasphemous sense, but in a truly Christian understanding of creating on behalf of God and so to continue God's initial process of creation as an image and proxy of God Himself (see Genesis 1:26–27).

**Figure 4:**
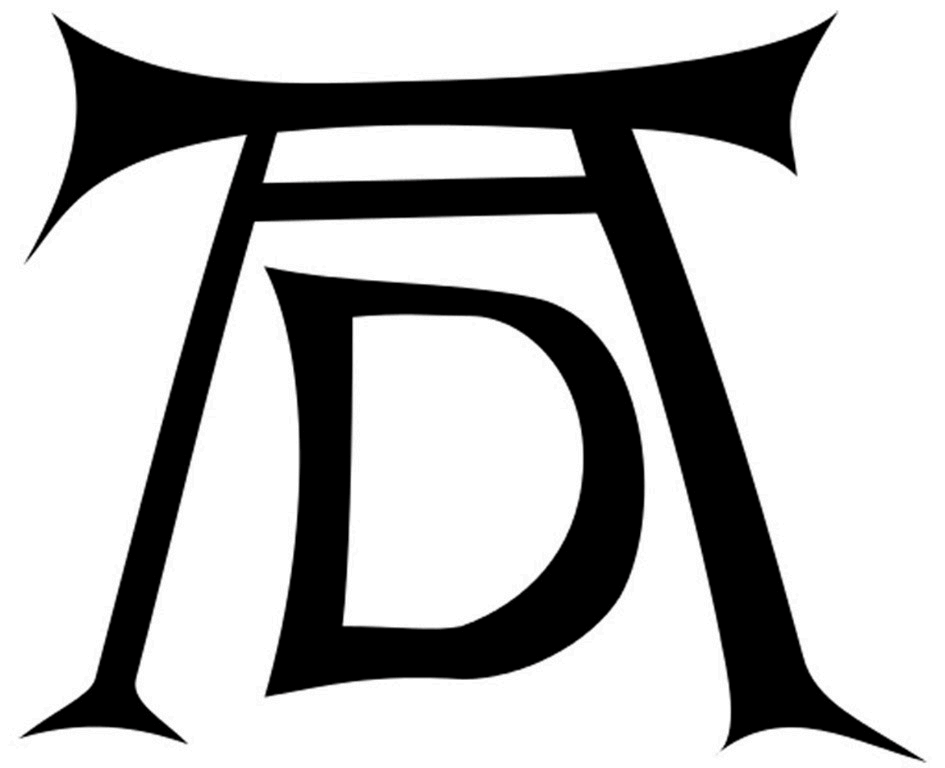
**Albrecht Dürer's monogram which he used from about the year 1497 on—the version here originates from 1498**. This work is in the public domain.

Dürer further intensified the message of his 1500 self-portrait by placing a clear indication of authorship in the main focus area of the painting at the eyes' level: to the left of his head, he positioned his signature, to the right, he further qualified that the portrait was about himself and that it was of eternal quality (“I, Albrecht Dürer of Nuremberg, portrayed myself in everlasting colors aged 28 years”; original Latin inscription: “Albertus Durerus Noricus/ipsum me propriis sic effin/gebam coloribus aetatis/anno XXVIII”). Signing a painting and referring to a specific artist was not very common in the time around 1500. Moreover, signing with a monogram that has the particular quality of Albrecht Dürer's one is even more a statement of the importance of the artist; Dürer designed one of the first corporate logos in world history with quite a simplistic, but highly recognizable and memorisable monogram just consisting of two letters: A and D (see Figure 4)—showing an interesting ambiguity as it stands for A[lbrecht] D[ürer], but also for A[nno] D[omini], the “year of the Lord.” Again, this points to a direct link between Dürer and Christ (Koerner, 1990).

Actually, the self-depiction of Dürer from 1500 became so popular and a common part of everyday culture that it even converted to a symbol and common representation of Christ himself: Out of a set of 160 historic depictions of Jesus Christ from the 4th to the 20th century plus the Dürer painting, naïve participants (*N* = 43) selected the Dürer as the most typical Jesus depiction (Carbon et al., 2010)—although Dürer's work did not really show Christ but only referred to the artist himself (note: many people do indeed interpret this work as depicting Christ, although the idiosyncratic and naturalistic outward appearance of Dürer becomes very clear)!

## Sujets, symbols and messages in selfie photography and artists' self-portraits

As noted above, self-portraits and selfies share the common ground of being born from the idea or wish to freeze a fluctuating but significant slice of life. Considering selfie photography, one will register that the appearance and mise-en-scène of such a slice of life can have a variety of faces. In other words: Selfie photography is quite multi-faceted and knows many different sujets, ranging from the “classic selfie” showing just one's own face over “Outfit selfies” to “AirSelfies” and so on. Table 1 sums up typical sujets or types of selfies together with the respective aims related to a specific sujet or type.

**Table 1:** **Overview of typical types of selfies, including a short characterization and main aims often found with people who take such selfies**.

**Type of selfie**	**Characterization**	**Main aims**
Classic selfie	Taking a photo just from the own face without more additional ingredients, looking quite neutral	• Self-reference • Documentation
Situation selfie	Portraying a specific situation in which the selfied person is currently (in the bed, in a miserable situation, with fun)	• Authenticity • Humor
Emotional selfie	Expressing a specific emotion very clearly and explicitly	• Emotion • Mood
Optimization selfie	Posing to optimize the physical appearance (e.g., by shooting from above, trimming the facial shape by muscle activities)	• Attractiveness • Idealization
Celebrity selfie	Integrating a celebrity while taking a selfie	• Importance • Identification
Sports selfie	Taking a selfie while making sports activities (indoor)	• Sportiveness • Energeticness • Performance
Leisure selfie	Taking a selfie being lazy, chilling out	• Mood
Food selfie	Selfie-ing while eating	• Authenticity • Passion
Drink selfie	Selfie-ing while drinking	• Authenticity • Passion
Mirror selfie	Shooting a selfie through a mirror	• Spontaneity • Authenticity
Landmark selfie	Posing in front of a significant landmark (building, landscape)	• Exclusivity • Interest
Outfit selfie	Focusing on new or special outfit	• Trendiness • Innovativeness
Body selfie	Pronouncing specific body parts, especially the belly (“belfie”), muscles, body parts of particular appeal or salience	• Sportiveness • Beauty Physical • properties
Car selfie	Taking a selfie while driving a car	• Spontaneity • Performance Personal • situation
Ultimate selfie/ Daredevil selfie	Initiating a stunt in the face of a camera	• Performance • Fearlessness
Purpose selfie	Making clear with the selfie that something important will go on (e.g., by showing a weapon, a claim of responsibility)	• Importance • Power
Fingermouthing selfie	Fingers are in front of the mouth or touch the lips	• Spontaneity • Expression • Attractiveness
Selfie-reference selfie	Making explicitly clear that the photo is a selfie by, e.g., shooting the selfie-ist in a mirror while making the selfie	• Self-reference • Creativity
Selfie-stick	Selfie taken from a farther distance as usual by help of a selfie-stick, a monopod which is typically extensible	• Context relationship • Part of the whole • Competence • Mastering of difficult situations
AirSelfie	Takes the selfie from a device that flies above the selfie-ist, mostly ensured by a camera drone	• Competence • Context relationship
Weefie	Shows not only the selfie-ist, but also other people who are directed toward the camera	• Social embedmen • Social relationship

Obviously any kind of self-portrait is a self-reference and is capable of documenting a certain moment of life. Selfies are often marked by the (additional) pretense of being authentic. So people depicted on selfies often make us believe that the photo was shot instantly and incidentally when the current situation emerged, although many situations are intuitively initiated just for the sake of making the selfie. Selfie-ists often want to convey a specific image of themselves, a rather euphemistic, self-serving image that is indeed far from authentic. Therefore, certain poses are trained to look slimmer or specific camera perspectives from above are utilized in order to suggest a lower weight (Schneider et al., 2012), for example. Pronouncing attractive or salient body parts can increase the impression of being healthy and sportive—a similar purpose can be identified when certain artifacts, actions, or contexts are used that are typically associated with these values and properties.

**Figure 5:**
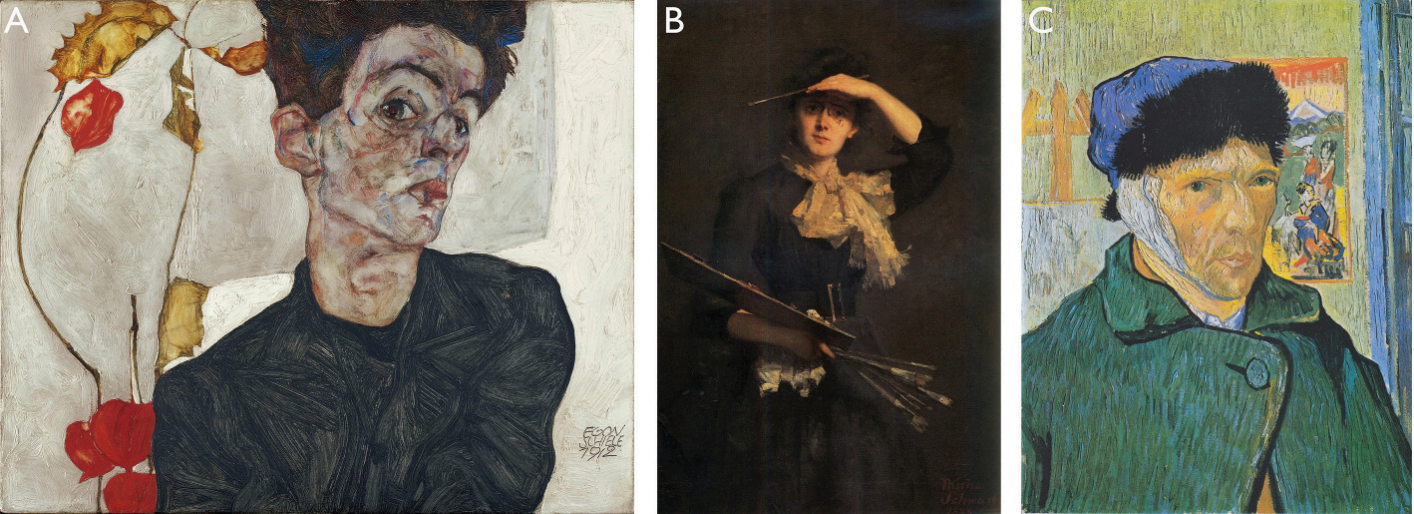
**(A)** Egon Schiele (1912): “Self-portrait with physalis—public domain, reproduction from The Yorck Project. **(B)** Thérèse Schwartze (1888): “Self-portrait with palette”—public domain. **(C)** Van Gogh (1889): “Self-portrait with bandaged ear” from 1889”—public domain. It is quite interesting to see what a reviewer of this manuscript has observed: Thérèse Schwartze holds the brush in the left hand while keeping the palette in her other hand. It is hard to find any notes on Schwarzte's handedness, but we know that dominance of a hand is an important personal and even identifying property of a painter, so it would be rather extraordinary if she had not compensated for the mirroring of the handedness by the mere usage of a mirror.

Systematizing the aims of the different sujets or types of selfies listed in Table 1, shows that these aims circle around three main factors: (A) self-expression, (B) documentation and (C) performance. Interestingly, these main message aims can also be found in painted self-portraits as I will show in the following by reference to several examples.

(A) Self-expression is, self-evidently, the core value of any self-portrait. Why should you make a portrait of yourself, if you did not want to express yourself? Self-expression is about the idea that the depicted person is different, unique, special in a sense of a personality trait—sometimes people just want to convey information on their current mood, or emotional and cognitive state. Certainly, painted self-portraits cannot provide many of such instances in life, just because creating paintings is effortful and expensive. Accordingly, they often refer to extraordinary, especially important or characteristic instances. For example, self-portraits may show or symbolize deep religious feelings, contemplation on something important or thinking about a special problem of the self-depicted artist. Other self-portraits, however, focus on certain fluctuating moments of life, or on expressing a certain mood like in Egon Schiele's “Self-Portrait with Chinese lantern lamp” from 1918 or his “Self-Portrait with physalis” from 1912 (see Figure 5A). As has already been shown, Dürer was a master of self-expression. Interestingly, he did not idealize his specific outward appearance in his self-portraits, but depicted it, contrary to the Italian Renaissance tradition (Koerner, 1990), in a literally hyper realistic way. What he indeed idealizes in expressing himself, is his meaning and special status. And this is not so different from many types of selfies where the protagonists try to emphasize or amplify some personality properties or situational specifics.

(B) Self-depictions also aim to document a certain status quo. In modern times this documentation is often realized by referring to certain achievements in a very explicit way, by use of paraphernalia or symbols: e.g., wearing a graduation cap indicates the success of graduation, showing specific artifacts can document a certain health status (e.g., a plastered arm, a crook), showing oneself next to one's crashed car indicates “hey, I survived,” and so on. In paintings we can see similar documentary attempts, although again the threshold for an event to be painted as a part of a self-portrait is of course much higher, e.g., having crossed the Alps in the case of Dürer's self-portrait from 1498 (see Figure 3B), or becoming a member of an exclusive circle, e.g., Thérèse Schwartze's “Self-portrait with palette” from 1888 (see Figure 5B), or being in a physically or psychologically extreme state like Van Gogh in “Self-portrait with bandaged ear” from 1889 after having cut a portion of his ear (see Figure 5C). Another extreme case is the “Self-portrait with the portrait of Dr. Farill” painted by Frida Kahlo in 1951 where she depicts herself being confined to a wheelchair—a painting which is also known as the last one she has ever signed.

(C) The aim of showing performance in a self-portrait is certainly tightly connected with the aforementioned categories, but focuses more strongly on capability and ability aspects of the artist. Paintings belonging to the performance category, besides some paintings portraying the artist in the state of painting, e.g., Van Gogh's “Self-Portrait in Front of the Easel” from 1888 (cf. type of selfie termed “Selfie-reference selfie” in Table 1), seem to be relatively rare in art history. Main reason for this might be the plain fact that the core performance painters show is painting, so the result of their work, the self-portrait, is often sufficient evidence for their performance already. There is an own sujet in art history showing also painters self-portraying themselves while painting: For instance, Velázquez depicting himself while painting the royal family in his masterpiece “Las Meninas” from 1656 or Vermeer's painting potentially portraying him from behind entitled “Art of painting” from 1666 (see Hall, 2014). Here we observe a painter in his studio who actually draws the model which is also depicted in the painting. Another example was created by René Magritte. In his “Attempting the impossible” (1928), he depicts himself while painting a female nude in life size—interestingly, this painting with this intriguing self-reference has itself been featured in the Belgian Surrealist journal Variétés with a selfie-like depiction of the artist in front of the painting, seemingly working on the painting. There are of course also some interesting self-portraits with other performance classes beside painting, e.g., Tamara de Lempicka's “Tamara in a Green Bugatti,” created in 1929, shows her driving fast in a sports car, or “Self-portrait with horn” (1938) where Max Beckmann paints himself as a musician. Other, even more sophisticated cases where “self-portraits” are composed in such a way that they show only some parts of the artist's body which would also be naturally be perceived when the correct perspective would be followed. Excellent examples for this perceptually highly interesting sub-group can be found among the works of Robert Pepperell who has created a couple of exemplars where he analyses his own perceptual conditions, for example, by showing the interior of the room plus the artists feet while lying on a chaise longue and drawing the interior of the room (Robert Pepperell: “Self view with feet after Mach,” painted in 2012).

## Coda: self-depictions as a compact format to communicate complex information

To sum up, although contemporary selfies are clearly produced with high frequency and often quite incidentally, they aim to provide similar messages and show similar types of expression as self-portraits from the domain of artistic painting did for centuries. They reveal something about the creator in particular, but also something about humans in general. Humans want to document their lives, their personality, their outward appearance, and sometimes also their current situation, their mood, feelings or cognition. This is also an expression of the social nature of the human being, wishing others to share one's experiences and to empathize with these experiences. To communicate this efficiently, statements are often enhanced. Self-portraits of any kind have to deliver these complex and multi-dimensional information in a very compact format, just in one single picture. That we still use such a simple format, although capturing dynamic scenes with modern multimedia-technique would be so easy by technical assistance today, is a sign for the adequateness and power of this format. Obviously, to generate one single picture, sometimes with all its inherent ambiguity, is an ideal way to provide a mixture of concreteness and imagination. On the one hand, transporting a very concrete depiction of oneself to document the current appearance and on the other hand, inviting the beholders to trigger their associations and imaginations to be personally touched and so to empathize with the creator.

## Author contributions

CCC made the research and wrote the entire paper.

### Conflict of interest statement

The author declares that the research was conducted in the absence of any commercial or financial relationships that could be construed as a potential conflict of interest.

## Footnotes

^1^One explanation for this low number is that Dürer was a very economically thinking painter: actually, prints of holy persons, e.g. of Virgin Mary, made much more profit than a singular self-portrait in oil (Hall, 2014)—still a few of these brilliant portraits showing the “artist behind the prints” were also important to underline the extraordinary quality of the creator.

^2^Probably, Kahlo was one of the most productive self-portraitists of all time as nearly half of her paintings were in fact self-portraits (i.e. 55 out of 143, Freeland, 2010).

^3^Art philosopher Freeland stresses this point, also by using an own word to describe the phenomenon that portraits convey “a person's very essence”: the person's “air” (Freeland, 2010, p. 44) which is very much based on Roland Barthes' reflections on photography published in his book “Camera lucida” (Barthes, 1981).

^4^Art philosopher Cynthia Freeland even adds this quality to the list of three requirements of a portrait: besides the physical delineation and conscious presentation of the self to be conveyed in the resulting artwork, she mentions the “indication of interior states” (Freeland, 2010, p. 17).

^5^Gombrich refers to e.g., Rembrandt as having portrayed himself with “great honesty” (p. 420).

^6^The painting is often misinterpreted as showing a man with a red turban. In fact, the depicted person wears a chaperon, which was a fashionable and prestigious hat worn in all parts of Western Europe in the times of Van Eyck.

^7^It should not be forgotten that despite these technical innovations, ancient mirrors were still relatively small (Melchoir-Bonnet, 2001), making it even more astonishing that painters like Dürer accomplished such large panels (in the case of the 1500 Pelzrock painting, e.g., 49 cm × 67 cm) of their own faces. Such sizes could only have emerged from a successive local painting strategy of the visual information conveyed by a small hand-held mirror.

^8^Newhall documented this case in the 1949 edition of his milestone work “The history of photography,” but he on his own was skeptical whether Cornelius was really the very first person who made a photographic self-portrait: “…one hesitates, in the absence of more complete documentation, to assign to any one of these pioneers the honor of priority” (Newhall, 1949, p. 24).

^9^Newhall already in 1937 mentioned that the specific use of specific lenses made it possible that Cornelius needed “only” five minutes in the reduced overall luminance conditions of October (Newhall, 1937).

## References

[B1] Alberti L. B. (1988). De re Aedificatoria on the Art of Building in Books. Transl. by Rykwert J. Tavernor R. Leach N. Cambridge, MA: MIT Press.

[B2] Barthes R. (1981). Camera Lucida in Reflections on Photography [La Chambre claire]. Transl. by Howard R. New York, NY: Hill and Wang.

[B3] Belle J. (2000). Five Hundred Self-Portraits. London: Phaidon Press.

[B4] Billeter E. (1986). Self-Portrait in the Age of Photography: Photographers Reflecting their Own Image. Houston, TX: University of Houston.

[B5] Bruno N. Gabriele V. Tasso T. Bertamini M. (2014). ‘Selfies’ reveal systematic deviations from known principles of photographic composition. Art Percept. 2, 45–58. 10.1163/22134913-00002027

[B6] Bulst N. Lüttenberg T. Priever A. (2002). Abbild oder wunschbild? bildnisse christoph ambergers im spannungsfeld von rechtsnorm und gesellschaftlichem anspruch. Saeculum 53, 21–74. 10.7788/saeculum.2002.53.1.21

[B7] Carbon C. C. Gruber P. Sommer P. (2010). On the search for the super-Jesus. which features does a depiction in art history need to be identified as Jesus? Perception 39:115.

[B8] Carbon C. C. Hesslinger V. M. (2015). Restoring depth to Leonardo's Mona Lisa. Am. Sci. 103, 404–409. 10.1511/2015.117.1

[B9] Eaton F. A. (1882). Albert Dürer, His Life and Works. London: J. Murray.

[B10] Freeland C. (2010). Portraits & Persons. Oxford: Oxford University Press.

[B11] Gigerenzer G. (2007). Gut Feelings: The Intelligence of the Unconscious. New York, NY: Viking Press.

[B12] Gombrich E. H. (2002). Art and Illusion. A Study in the Psychology of Pictorial Representation. London: Phaidon.

[B13] Gombrich E. H. (2005). The Story of Art, 16th Edn. London: Phaidon.

[B14] Hadsund P. (1993). The tin-mercury mirror: Its manufacturing technique and deterioration processes. Stud. Conserv. 38, 3–16. 10.1179/sic.1993.38.1.3

[B15] Hall J. (2014). The Self-Portrait: A Cultural History. London, UK: Thames & Hudson.

[B16] Hannavy J. (2005). Encyclopedia of Nineteenth-Century Photography. New York, NY: Routledge.

[B17] Harbison C. (1995). The Mirror of the Artist: Northern Renaissance Art and Its Historical Context, 1st Edn. New York, NY: Prentice Hall.

[B18] Hirsch R. (2000). Seizing the Light: A History of Photography. New York, NY: McGraw-Hill.

[B19] Kahneman D. (2003). Maps of bounded rationality: psychology for behavioral economics. Am. Econ. Rev. 93, 1449–1475. 10.1257/000282803322655392

[B20] Koerner J. L. (1990). Self Portraiture and the Crisis of Interpretation in German Renaissance Art: Albrecht Dürer, Hans Baldung Grien, and Lucas Cranach the Elder. Doctor of Philosophy Dissertation, University of California, Berkeley.

[B21] Melchoir-Bonnet S. (2001). The Mirror: A History. New York, NY: Routledge.

[B22] Newhall B. (1937). Photography 1839-1937. New York, NY: The Museum of Modern Art.

[B23] Newhall B. (1949). The History of Photography from 1839 to the Present Day. New York, NY: Castle Books.

[B24] Prakel D. (2009). The Visual Dictionary of Photography. Lausanne: AVA Publishing.

[B25] Sachse J. F. (1893). Share in the development of photography [A lecture delivered before the Franklin Institute, December 16, 1892]. J. Franklin Inst. State Pa. Promot. Mech. Arts 135, 271–287.

[B26] Schneider T. M. Hecht H. Carbon C. C. (2012). Judging body weight from faces: the height-weight illusion. Perception 41, 121–124. 10.1068/p7140 22611670

[B27] Tyler C. W. (1998). Painters centre one eye in portraits. Nature 392, 877–878.

[B28] Wade N. J. (2014). The first scientific “selfie”? Perception 43, 1141–1144. 10.1068/p4311ed 25638932

[B29] Wade N. J. (2016). Art and Illusionists. Cham: Springer.

[B30] Wegner D. M. (2003). The mind's self-portrait. Ann. N.Y. Acad. Sci. 1001, 212–225. 10.1196/annals.1279.011 14625362

[B31] Wheen A. (2011). DOT-DASH TO DOT.COM: How Modern Telecommunications Evolved from the Telegraph to the Internet. Heidelberg: Springer.

[B32] Zirpolo L. H. (2008). Historical Dictionary of Renaissance Art. Lanham: Scarecrow Press.

[B33] Zitzlsperger P. (2012). Dürers Pelz und das Recht im Bild, Kleiderkunde als Methode der Kunstgeschichte. Berlin: De Gruyter.

